# How to evaluate the impact of a citizen rescue system on survival from cardiac arrest?

**DOI:** 10.1007/s12471-019-1294-6

**Published:** 2019-05-29

**Authors:** P. Calle, N. Mpotos

**Affiliations:** 1 Emergency Department, General Hospital Maria Middelares, Ghent, Belgium; 2 Faculty of Medicine and Health Sciences, Ghent University, Ghent, Belgium; 3 Emergency Department, General Hospital Saint Lucas, Ghent, Belgium

Pijls et al. are to be congratulated on their analysis of the accuracy of the activation of a citizen rescue system for cardiac arrest [1]. Regarding their evaluation of the impact on survival, however, we would like to raise some methodological problems.

Based on a positive correlation between the density of volunteer rescuers and the gain in survival among victims with and without responding citizens (with an increase from 16.0% to 34.8% in the municipalities with the highest volunteer density), the authors state: ‘These findings suggest that survival may even further increase with higher numbers of volunteers’. However, the methodology used is inappropriate to prove causality between higher volunteer density and higher survival and to estimate the magnitude of the impact of a higher attendance rate of alerted citizen responders. Indeed, the reasons why some victims are not reached by the alerted volunteers and why some municipalities have a low volunteer density are not explored. Probably, age, co-morbidity, socio-economic status, distance to the nearest emergency medical services (EMS) station, location of the collapse, etc. play a role. As these factors have some prognostic value, the presence of an alerted volunteer at the scene may largely be a marker for good outcome, and not a pivotal element in survival. Consequently, the impact on survival of a high volunteer density may be less impressive than suggested by the findings of Pijls et al.

We believe that some answers to the issues we have raised may be provided by looking closely at the resuscitation measures taken by the alerted volunteers in all individual surviving patients [2]. Specifically, one needs to know if the alerted volunteers delivered automated external defibrillator (AED) shocks before EMS arrival, how many minutes elapsed between the delivery of these shocks and EMS arrival and how many minutes of basic life support (BLS) were provided by bystanders, on the one hand, and by alerted volunteers, on the other. Indeed, one should be aware that survival among the 79 survivors with at least one alerted volunteer might also have occurred without BLS and/or AED shocks delivered by these alerted volunteers. Quantification of the surplus value of the actions undertaken by the citizen rescue system in a particular patient will always be an estimate, but as a rule of thumb one may use a 10–12% decrease in survival rate for every minute of delay between the AED shock delivered by the alerted volunteer and EMS arrival. However, when BLS is provided, the decline in survival averages 3–4% per minute to defibrillation [3].

Fortunately, the authors gathered much information on each resuscitation attempt, including the actions taken by the alerted volunteers. This probably enables them to perform the calculations we propose in the 79 survivors with at least one alerted volunteer. We believe this is the most appropriate way to find out how many lives were saved by this novel citizen rescue system during a 2-year period in an area with 1.12 million inhabitants.
